# A Triazine Membrane for Sustainable Acquisition of Au(III) from Wastewater

**DOI:** 10.3390/molecules29092051

**Published:** 2024-04-29

**Authors:** Ge Shang, Haonan Dong, Yi Zhang, Conghuan Zhang, Ting Chen, Yunhua He, Hongxing He, Weili Li, Xiujun Deng, Zhifeng Nie, Sibiao Zhao

**Affiliations:** 1 Yunnan Key Laboratory of Metal-Organic Molecular Materials and Device, School of Chemistry and Chemical Engineering, Kunming University, Kunming 650214, China; 15516241842@163.com (G.S.); lierkm@kmu.edu.cn (W.L.); niezf123@kmu.edu.cn (Z.N.); 2 Kunming Institute for Food and Drug Control, Kunming 650034, China; 15887852700@163.com

**Keywords:** gold adsorption, selectivity, triazine membrane, phase transition method, DFT calculations

## Abstract

The recovery of Au(III) from solution using adsorbents in the form of granules or powders is challenging due to issues such as instability during the recovery process or mass loss caused by small particle size. This study introduces a PEI-TCT/PVDF composite membrane designed to intercept and capture Au(III) in wastewater. Experimental results demonstrated that the PEI-TCT/PVDF membrane exhibits a broad pH range (1–8) and a high retention efficiency for Au(III) of 97.8%, with a maximum adsorption capacity of 294.5 mg/g. The mechanism of Au(III) adsorption on the PEI-TCT/PVDF membrane was mainly through electrostatic adsorption, which caused AuCl_4_^−^ to aggregate on the surface of the membrane and gradually reduced to Au^0^ and Au^+^. Furthermore, the membrane can be entirely regenerated within 20 min and maintains its performance in subsequent adsorption cycles. This study highlights the potential of PEI-TCT/PVDF membranes for the recovery of precious Au(III).

## 1. Introduction

Gold is an important precious metal with significant economic value [1,2,3]. Recycling of gold-bearing secondary resources is of strategic importance. Not only can it alleviate the imbalance between supply and demand, but it can also meet the demand for strategic reserves. Current gold recovery methods include precipitation [4], ion exchange [5,6], solvent extraction [7,8,9], and adsorption [10,11,12,13]. Novel adsorbent materials such as biomass materials, mesoporous molecular sieves, metal–organic frameworks (MOFs) [14,15], and covalent organic frameworks (COFs) [16,17,18] have been successfully used in the adsorption recovery of Au(III). Lin et al. [19] reported thiosemicarbazide-functionalized corn bracts. It showed selective adsorption of Au(III) ions from an aqueous solution with a maximum adsorption capacity of 1470.22 mg/g. The polymer maintained high adsorption efficiency after three cycles (desorption time of 24 h). Yeung et al. [20] grafted amidopropyl and thiopropyl groups onto the surface of a mesoporous molecular sieve (MCM-41). The selective adsorption of gold (III) from aqueous solution was demonstrated. Li et al. [21] reported an MOF adsorbent (MIL-161) for Au(III) recovery. Its maximum adsorption capacity was 446.49 mg/g and the adsorption performance was stable after five cycles. Liu et al. [22] achieved selective adsorption and high-purity recovery of Au(III) by changing the structure of the covalent organic framework. This resulted in efficient recovery of gold ions with an adsorption capacity as high as 1834 mg/g. However, overcoming the difficulties in reclaiming due to instability caused by small particle size or mass loss remains challenging.

Compared with other methods, membrane separation is considered an effective and promising technique for gold ion adsorption due to its high efficiency, simple operation, easy recovery, energy savings, and environmental friendliness. Gold-containing wastewater typically has a low pH, making fluoropolymers, particularly polyvinylidene fluoride (PVDF), the preferred material for membrane production. PVDF offers advantages such as chemical stability, high-temperature stability, corrosion resistance, extreme mechanical strength, and processability. For example, Liu et al. [23] efficiently recovered Au(III) ions using a polymer-encapsulated membrane made of PVDF with a hydrophobic low eutectic solvent as the substrate. This method exhibits high reusability and stability, achieving 92.4% extraction efficiency after five consecutive experiments. Wang et al. [24] synthesized a thiourea-polyvinylidene fluoride (Th-PVDF) and cast it into an amphiphilic microporous membrane for Au(III) adsorption. The membrane demonstrated high selectivity for Au(III) and maintained its performance over four adsorption-regeneration cycles.

It is evident that derivatized PVDF-type microporous membranes exhibit superior performance in the adsorption and recovery of Au(III). However, anchoring more functional substances on PVDF membranes to enhance adsorption efficiency remains challenging. Polyethyleneimine (PEI), a cost-effective polymer with various primary, secondary, and tertiary amine groups, has shown promise in metal ion separation. The addition of PEI to adsorbents increases the binding sites on their surface and addresses the challenge of difficult recovery from water [25,26,27,28,29]. PEI cross-linked with 2,4,6-trichloro-1,3,5-triazine (TCT) enhances metal chloride anion adsorption due to increased N atoms and rigid rings [30,31]. Notably, PEI-TCT demonstrates a high adsorption capacity (1073.0 mg/g) and superior efficiency (95.6% in 10 s) for Au(III) recovery [32]. Therefore, in this study, PEI-TCT/PVDF composite membranes were developed using PVDF as the base membrane and modified with PVP, PEI, and TCT. These composite membranes were designed for the interception and capture of Au(III) from wastewater, aiming to increase adsorption active sites and facilitate the recovery of the powdered material from aqueous solutions.

## 2. Results

### 2.1. Optimization and Characterization of PEI-TCT/PVDF Membrane

#### 2.1.1. Optimization of PEI-TCT/PVDF Membrane

**Figure 1:**
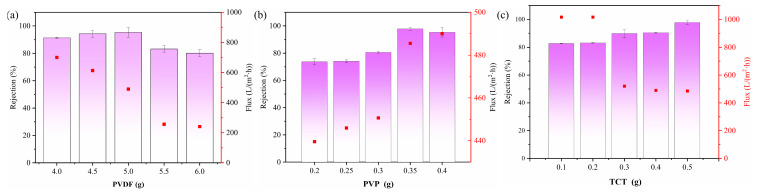
Optimization and characterization of PEI-TCT/PVDF membrane: (**a**) content of PVDF, (**b**) content of PVP, and (**c**) content of TCT in PEI.

In the conducted experiment, the influence of PVDF, PVP, and TCT content on the retention capabilities of PEI-TCT/PVDF membranes for Au(III) was systematically studied. As illustrated in Figure 1a, a non-linear trend was observed: Initially, the Au(III) retention increased with rising PVDF content, followed by a decline. Concurrently, the membrane flux exhibited a continuous decrease. This phenomenon can be attributed to the dual effect of PVDF on membrane properties. An increase in PVDF led to a reduction in pore size, reflected in lower flux, but excessive PVDF caused excessive wrapping of functional materials, thereby decreasing the surface area of exposed active sites per unit mass. Consequently, the best retention of Au(III) was achieved when the weight of PVDF was 5 g, striking a balance between retention and flux. Due to the hydrophobic nature of PVDF, we investigated the enhancement of its properties through the incorporation of PVP. Thus, the PVP content becomes pivotal in relation to PVDF. As illustrated in Figure 1b, the incorporation of PVP into PEI-TCT/PVDF membranes exerts a positive impact on the retention of Au(III), peaking at 0.35 g of PVP. However, beyond this threshold, retention diminishes. This decline could stem from the aggregation of PVP within the casting solution, reaching saturation as the concentration increases. Such saturation constrains the efficacy of PVP in bolstering retention, consequently resulting in a decrease in retention. TCT-PEI serves as a crucial adsorbent, exhibiting favorable interaction with Au(III). Nonetheless, an excessive quantity of TCT-PEI may adversely impact membrane formation. Figure 1c delineates the influence of PEI-TCT content on Au(III) adsorption. With the TCT concentration escalating from 0.1 g to 0.5 g, the retention of Au(III) on the PEI-TCT/PVDF membranes escalates to 82.7%, 83.1%, 89.8%, 90.3%, and 97.8%, respectively. However, when the TCT content exceeds 0.5 g, the film-forming ability is significantly reduced, and it is even difficult to find a film.

#### 2.1.2. Characterization of PEI-TCT/PVDF Membrane

**Figure 2:**
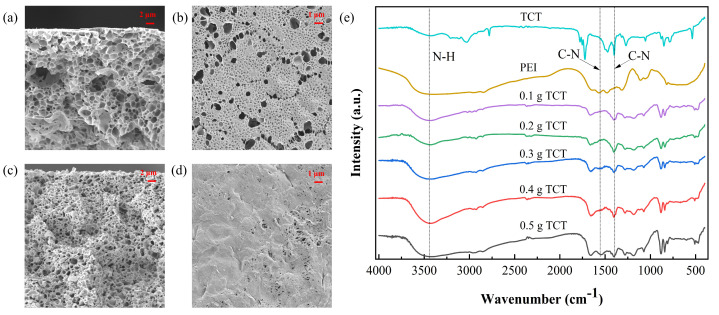
(**a**) SEM cross−section (0.1 g), (**b**) SEM cross−section (0.5 g), (**c**) SEM surface (0.1 g), (**d**) SEM surface (0.5 g), and (**e**) FTIR.

In Figure 2a–d, a substantial alteration in the membrane structure is evident as the TCT concentration rises from 0.1 to 0.5 g. This increase led to a transition from sparse to dense pores, resulting in a decline in pure water flux and a concurrent rise in Au(III) retention within the membrane. The FTIR spectrum of the PEI-TCT/PVDF membrane, as depicted in Figure 2e, displays a peak at 1397 cm^−1^, corresponding to the C-N backbone vibration of TCT. The N-H stretching vibration at 3417 cm^−1^ and the C-N stretching vibration at 1543 cm^−1^, attributed to the PEI component, confirm the successful incorporation of both PEI and TCT into the composite membrane.

### 2.2. Adsorption Studies of Au(Ⅲ) on PEI-TCT/PVDF Membranes

#### 2.2.1. Effect of pH on Membrane Performance

**Figure 3:**
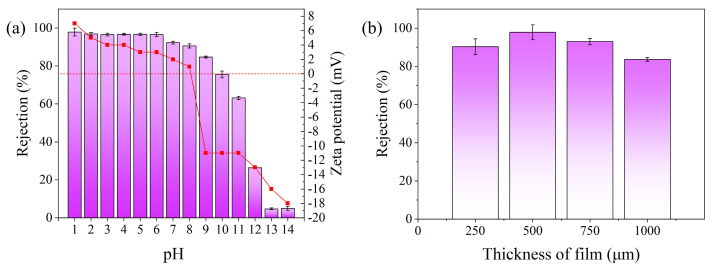
Adsorption studies of Au(III) on PEI−TCT/PVDF membranes: (**a**) pH and (**b**) thickness of the film.

The aim of this study was to investigate the effect of solution pH on the adsorption efficiency of Au(III) on the PEI-TCT/PVDF membrane. The pH was adjusted by using HNO₃ and NaOH in the range of 1–14. Adsorption tests (Figure 3a) showed that the adsorption efficiency was stable between 96.5% and 97.8% at pH values between 1.0 and 6.0. The adsorption efficiency gradually decreased as the pH value increased to 7.0. The adsorption efficiency reached 90.5% at pH 8.0. In the lower pH range (1.0–6.0), the adsorption efficiency remained about 96%. However, it decreased to 90.5% at pH 8.0 and further reduced to 84.6% at pH 9.0. Therefore, the optimum pH range for recovery of Au(III) through the PEI-TCT/PVDF membrane is between 1.0 and 6.0. The operating window at pH 8.0 is wider but less stable. This phenomenon can be attributed to the protonation of the imine/amine groups on the membrane at pH values below 8.1. This enhances the membrane adsorption by increasing the electrostatic interaction with the negatively charged ${\mathrm{AuCl}}_{4}^{-}$. Below pH 8.1, the membrane is positively charged, which enhances the adsorption capacity. At pH values above 9.0, the electrostatic interactions are weakened, leading to a decrease in the adsorption capacity. The adsorption capacity decreases at a pH value of about 8.0. This demonstrates the important role of imine/amine molecules in facilitating the rapid recovery of Au(III). This finding supports the idea that PEI-TCT/PVDF membranes can effectively recoat Au(III) by electrostatic adsorption. The proposed concept was verified.

#### 2.2.2. Effect of Membrane Thickness on Dynamic Recovery of Au(III)

Increasing the membrane thickness will correspondingly increase the adsorption active sites. The mass transfer region becomes longer, thus leading to longer penetration times. This method is typically used for dynamic adsorption separations to prevent premature permeation. In this experiment, an initial solution concentration of 20 mg/L Au(III) and a solution pressure of 0.08 MPa were used. The effect of membrane thickness on dynamic adsorption was investigated. Figure 3b displays the effect of membrane thickness on the dynamic adsorption of Au(III) recovery. The membrane retention efficiency increases and then declines with the increase in membrane thickness. The increase in membrane thickness extends the mass transfer region. This enhances the adsorption active sites and improves the membrane retention efficiency. However, the increase in membrane thickness also increases the mass transfer rejection. This leads to an increase in pressure drop and energy consumption of the whole system. This results in a decrease in membrane retention efficiency.

#### 2.2.3. Effect of Initial Solution Concentration on Dynamic Recovery of Au(III)

**Figure 4:**
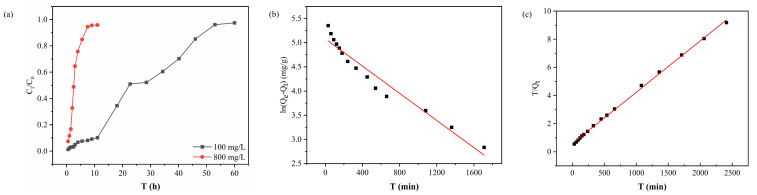
(**a**) Breakthrough curve, (**b**) pseudo-first-order mode, and (**c**) pseudo-second-order model fitting curves of PEI-TCT/PVDF membrane.

The effect of different concentrations of feed solutions on the retention process during dynamic adsorption is shown in Figure 4a. When the feed pressure was maintained at 0.08 MPa, the breakthrough curve changed significantly with the change in Au(III) concentration in the feed solution. The steepness of the breakthrough curve increased with increasing Au(III) concentration and the permeation rate accelerated. This phenomenon can be attributed to the increase in Au(III) concentration. More Au(III) ions occupy the limited number of adsorption active sites on the membrane per unit time. Therefore, as the initial concentration of Au(III) in solution increases, the time needed for the membrane to reach breakthrough decreases.

Figure 4b,c, along with Table 1, illustrate these observations. The equilibrium adsorption capacity (*Qe_1_*, cal) calculated using the pseudo-first-order model [33] is 160.37 mg/g, which differs notably from the experimental value (*Qe*, exp). The correlation coefficient R_1_^2^ was significantly low at 0.94876, indicating a poor fit of the data to the model. Conversely, employing the pseudo-second-order model yielded an equilibrium adsorption capacity of 273.97 mg/g. Its correlation coefficient, R_2_^2^, is significantly higher at 0.99871. This indicates a greater compatibility of the pseudo-second-order model with the experimental data. These findings suggest that the adsorption mechanism of the PEI-TCT/PVDF membrane aligns more closely with the pseudo-second-order model, indicative of a chemisorption mechanism.

### 2.3. Selectivity and Regeneration Experiments of PEI-TCT/PVDF Membrane on Au(III)

**Figure 5:**
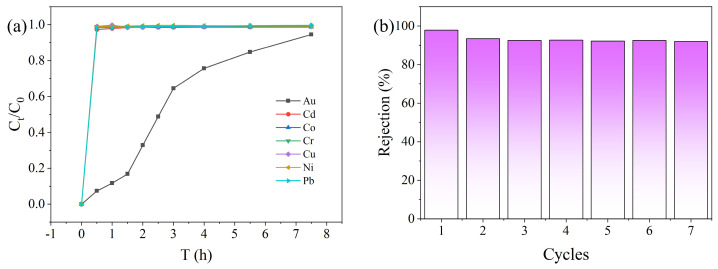
(**a**) Dynamic selectivity and (**b**) membrane recycling.

Maintaining sensitivity to Au(III) in complex water is a key property of PEI-TCT/PVDF membranes. The selectivity of PEI-TCT/PVDF membranes to Au(III) in mixed metal ion solutions was evaluated by filtration experiments. In order to simulate the actual conditions of the coexistence of Au(III) with a variety of other ions in water, a solution containing 20 mg/L each of Cu(II), Cr(III), Pb(II), Co(II), Cd(II), Zn(II), and Ni(II) and 800 mg/L Au(III) was prepared. The dynamic selectivity of PEI-TCT/PVDF membranes in the filtration process was studied. The results are presented in Figure 5a. Cu(II), Cr(III), Pb(II), Co(II), Cd(II), Zn(II), and Ni(II) rapidly penetrated into the membrane. A continuous breakthrough state is maintained. In contrast, the high concentration of Au(III) stayed in the membrane for a longer period of time. Table 2 summarizes the adsorption selectivity of PEI-TCT/PVDF membranes for Au(III) in the presence of coexisting metal ions. It is noteworthy that the partition coefficient of the PEI-TCT/PVDF membrane for Au(III) is 1573.08. However, the selectivity coefficients for competing metal ions range from 0.227 to 7.057. This indicates excellent adsorption selectivity.

In order to regenerate the adsorbent material, the membrane was treated with a desorption solution of 0.5 M thiourea/1 M HCl mixture. Thiourea is deemed to form complexes with metal ions and therefore effectively desorbs Au(III). As shown in Figure 5b, the system maintained good adsorption capacity and high adsorption rate after seven cycles. This indicates that the membrane material has good reusability and stability. This is critical for practical applications as it reduces the need for frequent replacement. The overall cost and environmental impact of the process were also minimized.

### 2.4. Adsorption Mechanism of PEI-TCT/PVDF Membrane on Au(III)

**Figure 6:**
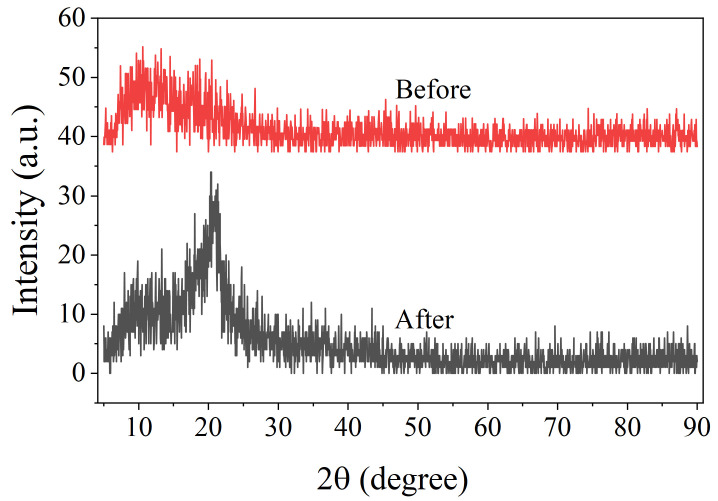
XRD of PEI-TCT/PVDF membrane.

The adsorption mechanism of Au(III) on PEI-TCT/PVDF membranes was elucidated. Emphasis was placed on the changes in chemical and structural properties before and after adsorption. XRD analysis in Figure 6 demonstrates that the initial membrane displays a broad and weak peak at about 2θ = 13°, indicating low polymer crystallinity. However, after adsorption of Au(III), a distinctly sharp peak appears near 20°. This is in agreement with the characteristic diffraction pattern of metallic gold. This suggests that some of the Au(III) was lowered to the metallic form during this process.

**Figure 7:**
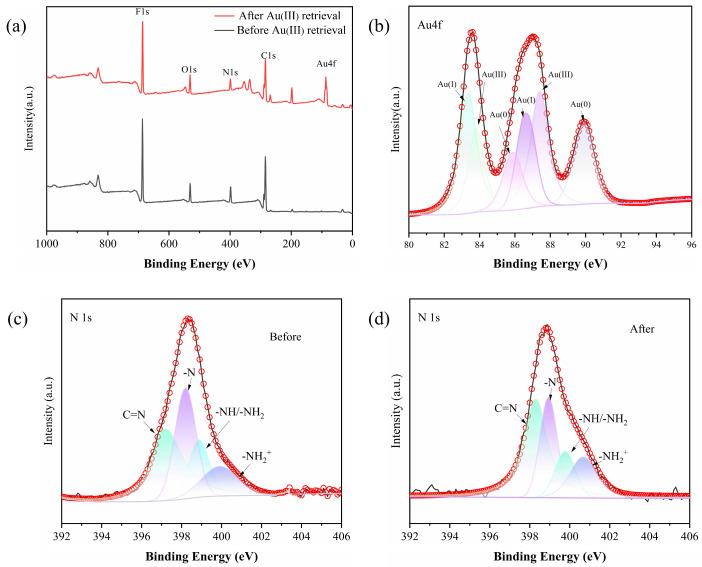
(**a**) XPS of PEI-TCT/PVDF membrane, (**b**) XPS(Au), (**c**) N1s before, and (**d**) N1s after.

The adsorption mechanism of Au(III) on the PEI-TCT/PVDF membrane was further elucidated using XPS spectroscopy (Figure 7a). Figure 7b illustrates that Au(III) adsorbed by the membrane was present in the forms of Au^0^ (with binding energies of 4f_7/2_ at 85.9 eV and 4f_5/2_ at 89.9 eV), Au^+^ (4f_7/2_ at 83.3 eV and 4f_5/2_ at 86.6 eV), and Au^3+^ (4f_7/2_ at 83.8 eV and 4f_5/2_ at 87.4 eV). The corresponding atomic ratios are 1:1.47:1.34. The existence of low oxidation states Au^0^ and Au^+^ suggests a chemical reduction process during adsorption.

The N1s spectra depicted in Figure 7c revealed nitrogen species in the PEI-TCT/PVDF membranes, including C = N (397.1 eV), -NH/-NH_2_ (398.9 eV), -N (398.2 eV), and $-{\mathrm{NH}}_{2}^{+}$ (399.9 eV). The atomic ratios are 1:0.82:0.41:0.38. Upon Au(III) adsorption, these peaks shifted to higher energies: 398.3, 399.7, 398.9, and 400.6 eV, indicating oxidation of the adsorbed gold ions. Additionally, the ratio altered to 1:0.54:0.30:0.42 (Figure 7d), demonstrating reduced percentages of -N and -NH/-NH_2_. This is consistent with the oxidation of nitrogen sites after the chemisorption of chelated Au(III).

**Figure 8:**
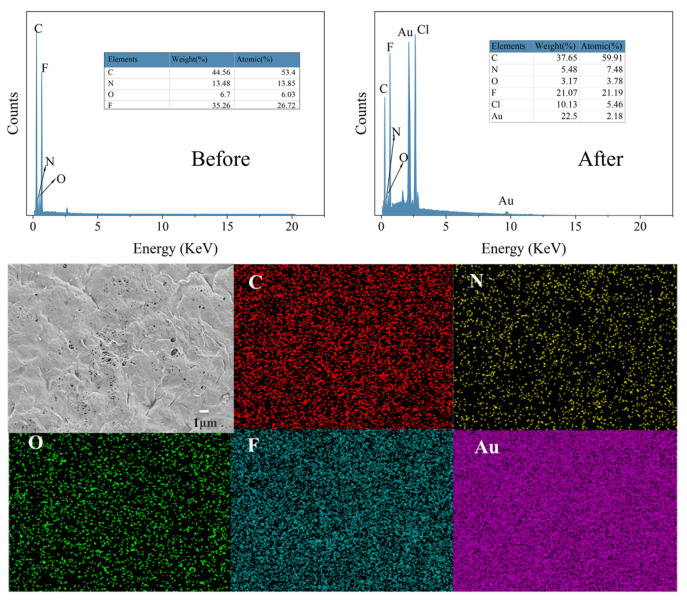
The EDS and mapping analysis of the PEI-TCT/PVDF membrane surface.

The EDS analysis of the PEI-TCT/PVDF membrane surface before and after the adsorption of Au(III) was conducted, and the results are presented in Figure 8. The figure clearly shows a significant increase in the content of the Au peak at 2.2 and the Cl peak at 2.7 after adsorption. This suggests that ${\mathrm{AuCl}}_{4}^{-}$ is adsorbed on the PEI-TCT/PVDF membrane surface through electrostatic adsorption. Additionally, the Au peaks between 8.5 and 13.5 indicate that Au(III) undergoes a redox process on the PEI-TCT/PVDF membrane surface. On the one hand, the positively charged amine functional groups on the surface of the PEI-TCT/PVDF membrane attract and adsorb ${\mathrm{AuCl}}_{4}^{-}$ from the solution electrostatically. Subsequently, a portion of the adsorbed ${\mathrm{AuCl}}_{4}^{-}$ is gradually reduced to Au^0^ and Au+. This finding is consistent with previous XPS results. Mapping analysis further confirmed that the Au element is bound to the PEI-TCT/PVDF membrane surface.

The adsorption sites were identified using the Fukui function of DFT calculation. The f_A_^+^ of the adsorbent was calculated by the following formula. The value of f_A_^+^ represents the degree to which the region is attacked by nucleophiles. A high ability to be attacked by nucleophiles indicates that there are sites in the adsorbent that can accept electrons. The part should be a positively charged region to be in a position to accept negatively charged ${\mathrm{AuCl}}_{4}^{-}$.

$$f_{A}^{+}=q_{N}^{A}-q_{N+1}^{A}$$

where $q_{N}^{A}$ represents the state of the adsorbate in which no electrons are gained or lost, and $q_{N+1}^{A}$ represents the state of the adsorbate in which e^−^ is gained.

**Figure 9:**
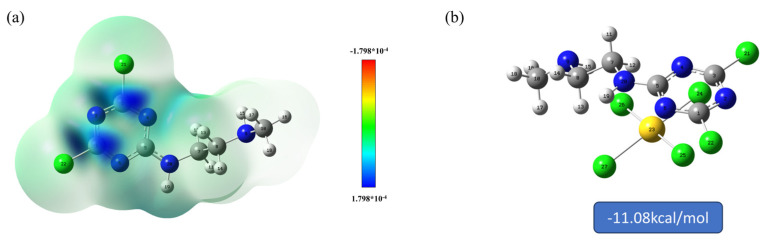
(**a**) Visualization of the Fukui’s function and (**b**) binding energy with ${\mathrm{AuCl}}_{4}^{-}$.

In Table 3 and Figure 9a, it can be seen that the parts susceptible to nucleophilic attack are mainly related to C and N in the triazine ring, where the positions C1, C3, N3, and N6 are more susceptible to attack by nucleophilic ${\mathrm{AuCl}}_{4}^{-}$. The binding energy of the adsorbent to ${\mathrm{AuCl}}_{4}^{-}$ was also calculated using DFT to be −11.08 kcal/mol (Figure 9b), indicating that there is a good binding between the adsorbent and ${\mathrm{AuCl}}_{4}^{-}$, which is favorable for adsorption to occur. This is in keeping with the analytical results of the Fukui function.

**Figure 10:**
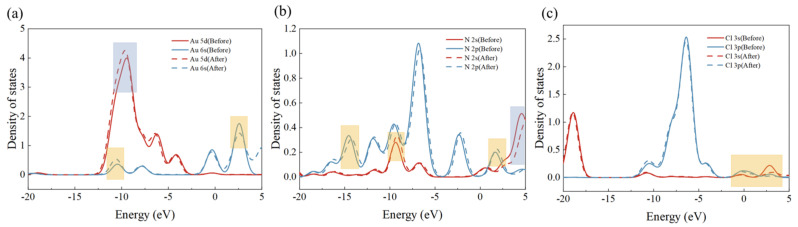
The partial state densities for the following species are presented: (**a**) Au, (**b**) N, and (**c**) Cl.

A wave function partial density of states (PDOS) analysis was conducted to investigate the electron transfer process during adsorption. The images demonstrate that the density state of the adsorbed Au 5 d orbitals is significantly enhanced at 10 eV and the Au 6 s orbitals exhibit a slight enhancement and weakening around −12 eV and 2.5 eV, respectively (Figure 10a). The density of states of the 2 s orbitals of N is enhanced and weakened around −9 eV and 4 eV, respectively, with significant shifts. In addition, the 2 p orbital of N is also significantly shifted around −14 eV (Figure 10b), and the changes in the 3 s and 3 p orbitals of Cl are mainly concentrated in the range of −2 to 5 eV, which are weakened to different degrees (Figure 10c). The combination of the above indicates that there is a significant electron transfer between the adsorbent and ${\mathrm{AuCl}}_{4}^{-}$. By combining the wave function analysis of the adsorbent and ${\mathrm{AuCl}}_{4}^{-}$ with the prediction of its charge transfer, it can be determined that the number of electrons gained by ${\mathrm{AuCl}}_{4}^{-}$ during the entire process of adsorption is −0.1485 eV. This indicates that ${\mathrm{AuCl}}_{4}^{-}$ transfers electrons it carries to the adsorbent by 0.1485 eV. This result is consistent with the PDOS presented in the previous section. In conclusion, it can be stated that the adsorption process between the adsorbent and ${\mathrm{AuCl}}_{4}^{-}$ is accompanied by charge transfer and there is chemisorption.

## 3. Materials and Methods

### 3.1. Chemicals and Materials

Polyethyleneimine (PEI) (M.W. 10,000, 99%) and 2,4,6-trichloro-1,3,5-triazine (TCT) (99%) were purchased from Shanghai McLean Biochemical Science and Technology Co., Ltd. (Shanghai, China). Tetrahydrofuran (THF, 99%) and potassium dichromate (K_2_Cr_2_O_7_, AR) were supplied by Chengdu Colony Chemical Company (Chengdu, China); nickel nitrate (Ni(NO_3_)_2_·6H_2_O, AR), lead nitrate (Pb(NO_3_)_2_, AR), copper nitrate (Cu(NO_3_)_2_·3H_2_O, AR), zinc sulfate (NiSO_4_·7H_2_O, AR), cobalt nitrate (Co(NO_3_)_2_·6H_2_O, AR), and cadmium nitrate (Cd(NO_3_)_2_·4H_2_O, AR) were purchased from the Beijing Chemical Factory (Beijing, China); polyvinylidene fluoride (PVDF) (Kynar Flex 2801), polyvinyl pyrrolidone (PVP) (K30), and HAuCl_4_‧4H_2_O (AR) were supplied by Sinopharm Chemical Reagent Co. (Beijing, China). All reagents are of analytical grade and used without further purification. All solutions are prepared with ultrapure water (18.25 MΩ·cm).

### 3.2. Instruments and Equipment

The XPS measurements were carried out on an ESCALAB 250Xi spectrometer (Thermo Scientific, Waltham, MA, USA) equipped with a pass energy of 30 eV with a power of 100 W (10 kV and 10 mA) and a mono-chromatized AlKα X-ray (hν = 1486.65 eV) source. All samples were analyzed under a pressure of less than 1.0 × 10^−9^ Pa. Spectra were acquired through the advantage software (Version 5.979) with a step of 0.05 eV. Ultra-sonic cleaning was performed in a bench top SK series ultrasonic cleaner (Shang-hai KODA Ultrasonic Instrument Co., Ltd., Shanghai, China), which operates at 35 kHz/53 kHz, power supply: 220 V/50 Hz, ultrasonic (W): 500. XRD measurements were performed on a Bruker D2 PHASER (USA) using a diffractometer with a copper X-ray source, a standard ceramic X-ray tube and a monochromator, a Kα filter, and an ROI. 2θ was evaluated over a range of 5–90° with a step size of 0.025. The application parameters for the copper lamp were 30 kV and 10 mA. SEM/EDS images were obtained using a high-resolution field emission scanning electron microscope (Hotfield Zeiss MERLIN Compact, S4800, Hitachi, Tokyo, Japan. Tokyo, Japan). Samples were analyzed in the 450–4000 cm^−1^ range using a Fourier transform infrared spectrometer (640-IR, Agilent Technologies, Santa Clara, CA, USA).

### 3.3. Preparation of PEI-TCT/PVDF Membranes

PEI-TCT/PVDF membranes were synthesized using phase change technology. The manufacturing process consisted of dissolving PEI (2 g)-TCT (0.1–0.5 g) in a 50 mL round bottom flask. It is mixed with a predetermined proportion of DMF (25 mL) and specific concentrations of PVP (0.2–0.4 g) and PVDF (4–6 g). The mixture is heated to 70 °C and stirred continuously at this temperature for 3 days to form a casting solution. The solution was degassed using ultrasound to eliminate air bubbles. The solution is then poured onto a flat glass surface and spread to form a film. It was immersed in water at 25 °C to complete the phase change and form the PEI-TCT/PVDF film. By adjusting the levels of PVP, PVDF, and PEI-TCT, different film properties can be obtained.

### 3.4. Experimental Method

**Figure 11:**
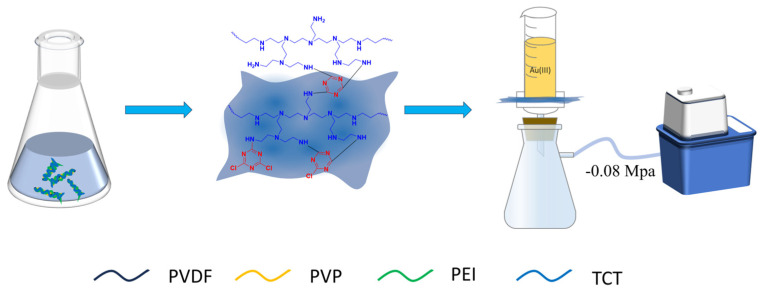
Filtration system for PEI−TCT/PVDF membranes.

The schematic diagram of the filtration system for PEI-TCT/PVDF membranes is illustrated in Figure 11. The PEI-TCT/PVDF membrane was positioned on a 2 cm effective diameter filtration flask, and the filtrate was collected at regular intervals using a negative pressure of 0.08 MPa as the driving force. The concentration of metal ions was determined using ICP-OES. Membrane flux and retention tests were conducted using ultrapure water and an Au(III) solution, respectively. Membrane flux (*J*), rejection (*R*), partition coefficient (*D*), and selection coefficient (*k*) were calculated by Equations (1), (2), (3), and (4), respectively.

(1) $$J=\frac{V}{T\times A}\times 100\%$$

(2) $$R=\frac{C_{0}-C_{t}}{C_{0}}\times 100\%$$

(3) $$D=\frac{\left(C_{0}-C_{e}\right)}{C_{e}}\times \frac{V_{1}}{W}$$

(4) $$k=\frac{D_{T}}{D_{M}}$$

(5) $$ln\left(Q_{e}-Q_{t}\right)=lnQ_{e}-k_{1}t$$

(6) $$\frac{t}{Q_{t}}=\frac{1}{k_{2}Q_{e}^{2}}+\frac{1}{Q_{e}}t$$

where *J* (L·m^−2^·h^−1^) represents the membrane flux; *V* (L) represents the total volume of sampling; *T* (h) denotes the total time of sampling; *A* (m^2^) represents the effective area of the membrane; *R* (%) represents the retention rate; *C_t_* (mg/L) represents the concentration of the solution at the moment t; *C*_0_ (mg/L) represents the initial concentration of the solution; *C_e_* (mg/L) represents the equilibrium concentration of the solution; *V*_1_ (mL) represents the volume of solution; *W* (g) represents the mass of the membrane; *D_T_* represents the partition coefficient of Au(III) and DM represents the partition coefficient of the competing ions in the solution; *Q_e_* (mg/g) represents the amount adsorbed at equilibrium; *Q_t_* (mg/g) represents the amount adsorbed at time t; and k_1_ represents the pseudo-first-order mode adsorption rate constant; k_2_ represents the pseudo-second-order model adsorption rate constant.

### 3.5. DFT Calculation

The DFT calculations in this paper were performed using Gaussian16 software [34]. The structures are fully optimized at the BP86-D3(BJ)/def2-SVP level, which combines the gradient-corrected BP86 functional [35,36] with the BJ-damped dispersion correction [37] and the def2-SVP basis set [38,39], using the SMD [40] solvation model for water solvent. The optimized structures are characterized by frequency analysis at the same level. The geometrically optimized structures were then adopted for single-point energy calculations at the bp86-D3(BJ)/def2-TZVP level. The wave function analysis was calculated by means of the Multiwfn version 3.8 (dev) code [41,42]. Finally, adsorption energy (E_ad_) calculations were carried out between adsorbates and Au(III) using Equation (7).

(7) $$E_{ad}=E_{AuCl_{4}-adsorbates}-E_{AuCl_{4}^{-}}-E_{adsorbates}$$

where $E_{AuCl_{4}-adsorbates}$ is the total energy of ${\mathrm{AuCl}}_{4}^{-}$ complexed with adsorbates; $E_{AuCl_{4}^{-}}$ denotes the energy of ${\mathrm{AuCl}}_{4}^{-}$; and $E_{adsorbates}$ is the total energy of the adsorbates.

### 3.6. Preparation of the Solution

The solutions of Au(III) and the rest of the metal ions used in the experiments were taken by dissolving the corresponding solids and diluting them using ultrapure water. The preparation of Au(III) solution was considered as an example. Firstly, an Au(III) stock solution with a concentration of 1913 mg/L was prepared by dissolving 1 g of HAuCl₄·4H₂O in ultrapure water. The stock solution was then continued to be diluted with ultrapure water to prepare 800, 200, and 100 mg/L Au(III) solutions. The additional metal ion solutions with 20 mg/L were prepared similarly to the above procedure.

## 4. Conclusions

In this paper, a PEI-TCT/PVDF composite membrane was designed with PVDF as the base membrane, and it was modified with PVP, PEI, and TCT to prepare a PEI-TCT/PVDF composite membrane for dynamic adsorption and retention of Au(III) in wastewater. The membrane composition and thickness, solution initial concentration, and pH were optimized. The PEI-TCT/PVDF composite membrane was able to retain Au(III) up to 294.5 mg/g. Dynamic wastewater tests showed that the membrane had good selectivity for Au(III) and maintained good stability and repeatability, as well as high adsorption capacity, after 7 regeneration cycles. XRD, XPS, EDS characterization, and DFT theoretical calculations showed that the mechanism of Au(III) adsorption on PEI-TCT/PVDF membranes mainly involves aggregating ${\mathrm{AuCl}}_{4}^{-}$ on the membrane surface through electrostatic adsorption initially. After that, it was gradually reduced to Au^0^ and Au^+^.

## Figures and Tables

**Table 1:** Adsorption kinetics parameters of PEI-TCT/PVDF membrane.

Adsorbent	*Q_e_* _exp_ /(mg/g)	Pseudo-First-Order Model			Pseudo-Second-Order Model		
		K_1_/min	*Q_e_*_1 cal_ /(mg/g)	R_1_^2^	K_2_/min	*Q_e_*_2 cal_ /(mg/g)	R_2_^2^
PEI-TCT/PVDF	294.5	0.0014	160.37	0.94876	0.00365	273.97	0.99871

**Table 2:** Adsorption selectivity of PEI-TCT/PVDF membranes for Au(III) in coexisting ionic systems.

Metalions	Au(III)	Ni(II)	Co(II)	Cu(II)	Zn(II)	Pb(II)	Cd(II)	Cr(III)
D/(mL/g)	1573.08	0.227	2.189	1.728	2.547	7.057	4.643	0.941
k	1	6927.85	718.65	910.29	617.74	222.89	338.84	1672.28

**Table 3:** Correlated calculated values of the Fukui function for sites susceptible to nucleophilic (f_A_^+^) attack by adsorbates. Visualization of the Fukui’s function in Figure 9a.

Number	Atom	f_A_^+^
1	C	0.157
2	N	0.035
3	C	0.168
4	N	0.149
5	C	0.011
6	N	0.157
7	C	−0.004
9	N	0.002
11	H	0.014
15	H	0.002
16	H	0.002
20	N	0.045
21	Cl	0.108

## Data Availability

The data are contained within the article.
